# Epidemiology of patients who died in the emergency departments and need of end-of-life care in Korea from 2016 to 2019

**DOI:** 10.1038/s41598-023-27947-z

**Published:** 2023-01-13

**Authors:** Sun Young Lee, Young Sun Ro, Sang Do Shin, Eunsil Ko, Seong Jung Kim

**Affiliations:** 1 Public Healthcare Center, Seoul National University Hospital, Seoul, Korea; 2 Laboratory of Emergency Medical Services, Seoul National University Hospital Biomedical Research Institute, Seoul, Korea; 3 Department of Medicine, Seoul National University college of Medicine, Seoul, Korea; 4 Department of Emergency Medicine, Seoul National University Hospital, 101 Daehak-Ro, Jongno-Gu, Seoul, 03080 Korea; 5 Department of Emergency Medicine, Seoul National University College of Medicine, Seoul, Korea; 6 National Emergency Medical Center, National Medical Center, Seoul, Korea; 7 Department of Emergency Medicine, Chosun University Hospital, 365 Pilmun-Daero, Dong-Gu, Gwangju, 61453 Korea

**Keywords:** Health care, Palliative care

## Abstract

The need of palliative care at the end-of-life in the emergency departments (ED) is growing. The study aims to investigate the epidemiology of patients who died during care in ED using nationwide database, and to estimate the need for palliative care in the ED. A retrospective observational study was conducted using the National Emergency Department Information System (NEDIS) database. Patients who died during ED care between 2016 and 2019 were included. Palliative care-eligible disease was defined as cancer (C00–C99 of ICD-10), chronic respiratory disease (CRD, J44–J46), chronic liver disease (CLD, K70–K77), and heart failure (HF, I50). Among the 36,538,486 ED visits during 4 years, 34,086 ED deaths were included. The crude incidence rate of ED deaths per 100,000 person-year was steady between 16.6 in 2016 and 16.3 in 2019 (p-for-trend = 0.067). Only 3370 (9.9%) ED deaths were injury, while 30,716 (90.1%) deaths were related to diseases. The most common ED diagnosis was cardiac arrest (22.1%), followed by pneumonia (8.6%) and myocardial infarction (4.7%). In cases of disease-related ED deaths, about 34.0% stayed longer than 8 h in the ED (median (interquartile range): 4.5 (1.9–11.7) h) and 44.2% received cardiopulmonary resuscitation (CPR) at end-of-life time. A quarter of the disease-related ED deaths were diagnosed with palliative care eligible disease: cancer (16.9%), CLD (3.8%), HF (3.5%), and CRD (1.4%). Cancer patients received less CPR (23.4%) and stayed longer in the ED (median (interquartile range): 7.3 (3.2–15.9) h). Over the past 4 years, more than 30,000 patients, including 5200 cancer patients, died during care in the ED. A quarter of disease-related ED death were patients with palliative care-eligible condition and more than 30% of them stayed longer than 8 h in the ED before death. It is time to discuss about need of palliative care in the ED.

## Introduction

The importance of palliative and end-of-life care is emphasized^1–3^ to an extent where the World Health Organization (WHO) recommends providing palliative care for most incurable patients and recognizing end-of-life care as an ethical obligation^3,4^. This is, however, still in an introductory stage and is provided only in limited places and to limited subjects^5^. In Korea, the need for palliative and end-of-life care is increasing after the enactment of “Act on Hospice and Palliative Care and Decisions on Life-Sustaining Treatment for Patients at the End-of-Life” (hospice act) in 2018^6^.

The emergency department (ED) is a specialized space for treating acute medical problems and saving lives, and therefore mortality rate in the EDs is relatively higher than that of general wards^7^. Some people die in ED from results of unexpected events or from sudden development of acute illnesses such as acute myocardial infarction or traumatic brain injury, while some patients with chronic diseases visit the ED to control rapidly worsening symptoms and serious complications of treatment and die in the ED^8,9^. Death during emergency care in the ED can be unavoidable for critically ill patients; however, there are little information about how many patients and who died in the ED^10^.

As prevalence of the advanced disease increases, there is an increase in ED visits at end-of-life for patients with terminal cancer or chronic illnesses^8,9,11–13^. People in proximity to death use more acute healthcare services than people who are not^14^. Among them, patients diagnosed with cancer and respiratory diseases are more likely to use acute-based healthcare service such as ED^15^. More than half of elderly patients who passed away in the ED had serious chronic illnesses and required palliative care^16,17^. Many ED visits in these terminally ill patients are considered avoidable, therefore, the need for palliative care at the end-of-life in ED is emphasized in many countries^18,19^. In the Unites States, there is a guideline for hospice care-eligible patients who visit the ED, and ED-initiated palliative care is recommended for patients with advanced cancer to improve the patients’ quality of life^20,21^. United Kingdom also reported end-of-life care recommendations for dying patients in the ED^22^. However, there is currently insufficient information on how patients who need end-of-life care are being treated in the ED and how many patients with incurable diseases received life-sustaining treatment such as cardiopulmonary resuscitation (CPR) in the ED before death. To improve the palliative care at end-of-life for terminally ill patients, it is necessary to understand the epidemiologic characteristics of patients who died in the ED^18,23^.

The purpose of this study is to investigate the epidemiologic characteristics of patients who died in the ED using representative nationwide database and to identify the reasons of ED visits of those patients. The study intends to estimate the need for palliative care at end-of-life in the ED and to accumulate the baseline data for future research.

## Methods

### Study design and data sources

This is a retrospective observational study using data from the National Emergency Department Information System (NEDIS) database. The Ministry of Health and Welfare constructed this nationwide ED-based database in 2013. NEDIS collected administrative and clinical information of all patients who visited EDs in real time from a total of 402 EDs across the country in 2019^24–26^. The information includes patient demographics, prehospital, and ED treatment. All patient-related information was automatically transferred from each ED to a central government server within 14 days of discharge from the ED or hospital. The trained coordinators designated in each institution managed the data uploading process. The NEDIS data is updated by the National Emergency Medical Center and is approved annually by National Statistics for data quality management.

### Study setting

Korea has a total population of 50 million, while the annual death rate was about 0.3 million in 2019. More than 70% of deaths occurred in medical institutions, and 14% at homes. The most common causes of death were cancer, heart disease, and pneumonia^27^.

The National Health Insurance (NHI) of Korea covers the entire population for inpatient, outpatient, and ED treatment. EDs are classified into 3 levels according to resources and capacity (including facilities, equipment, and medical staff), which are defined by the Ministry of Health and Welfare: 38 Level 1 EDs, 125 Level 2 EDs, and 239 Level 3 EDs (a total of 402 EDs), that are operational as of 2020. Level 1 ED provide the highest level of emergency care services in the country, and is designed to accommodate and to provide definite care to critically ill and severe emergency patients. Level 1 and 2 are emergency centers which take charge of treating critical ill patients, and Level 3 is in charge of general urgent care and primary responses 24 h a day, 7 days a week. Under this healthcare system, EDs are open to all beneficiaries without restriction, and patients have high accessibility to the ED care^28^.

Hospice and palliative care was first introduced to Korea in 2015 with inpatient hospice beds. Consultation-based hospice care for non-hospice wards and outpatients started as a pilot project in 2017 and home-based hospice care was officially started in 2020^29^. The hospice act defined four disease groups as target disease for palliative care: cancer, Acquired Immunodeficiency Syndrome (AIDS), chronic respiratory disease, and chronic liver cirrhosis as target disease for palliative care^6^. However, it is still in the introductory stage; there are not many providers of hospice ward and home-based hospice. The consultation-based hospice is mainly for inpatients and outpatients, and rarely applied to patients in ED.

### Study population

The study subject were patients who died during care in the ED (hereafter, ED deaths) in Korea for 4 years from January 2016 to December 2019. Death on arrival (DOA), patients who had already had cardiac arrest at ED arrival and did not receive any resuscitation efforts, patients who visited ED for non-medical reasons such as issuance of medical certificates, and patients who had unknown information of age or reason for ED visit were excluded.

### Variables and measurements

Based on the reason for ED visit, all ED deaths were initially classified into a disease-related group and injury-related (injury) group. For disease-related ED deaths group, the specific reasons for ED visits were classified based on the ED diagnosis codes of the international classification of diseases, 10th edition (ICD-10) in the ED discharge records written by emergency physicians.

Palliative care-eligible disease was defined based on the hospice act and literature review: cancer, chronic respiratory disease, chronic liver disease, and heart failure^30^. Palliative care-eligible disease was defined based on the ED diagnosis: cancer (ICD-10 code, C00–C99), chronic respiratory disease (chronic obstructive pulmonary disease and asthma, J44–J46), chronic liver disease (K70–K77), and heart failure (I50).

The following variables were collected from the NEDIS database to investigate the characteristics of ED deaths: 1. patient demographics (age, sex, and insurance), 2. prehospital and ED information (date and time of ED visit, mode of ED visit (use of ambulance or not), route of ED visit (direct visit or transfer-in from other hospital), region of ED (metropolitan or urban/rural), and level of ED (level 1, 2 and 3)), 3. care and outcomes (mental status at ED entrance, ED length of stay (ED-LOS), life-sustaining treatment (CPR and endotracheal intubation), ED diagnosis (ICD-10 code, multiple choice up to 20 codes), ED disposition (place, date and time of disposition), and ED death)^31,32^.

### Statistical analysis

A descriptive analysis was conducted to investigate epidemiologic characteristics of the study population. Categorical variables were presented as counts and proportions and differences across groups were tested by Chi-square test. Continuous variables were presented as medians and interquartile ranges (IQR) and differences across groups were tested by Wilcoxon rank-sum test.

The characteristics were compared according to the reason for ED visit (disease-related and injury-related). The most common diagnoses were investigated for the disease-related ED deaths group and cancer-related ED deaths.

The epidemiologic trends of all ED deaths and ED deaths of patients with palliative care-eligible conditions were investigated. The crude and the age- and sex- standardized incidence rates per 100,000 person-years were calculated. Direct standardization method was used using the 2020 mid-year census population as the standard population^33^.

NEDIS database collected during 2016–2019 were analyzed in July 2021. All statistical analyses were conducted using SAS software version 9.4 (SAS Institute Inc., Cary, NC, USA). Statistical significance was taken as P < 0.05.

### Ethical statement

This study was approved by the Institutional Review Boards of Seoul National University Hospital (approval No. SNUH-2012–104-1183), and the requirement for informed consent was waived due to the retrospective nature of this study. Patient information was anonymized prior to analysis. All methods were performed in accordance with relevant guidelines and regulations.

### Patient and public involvement statement

The National Emergency Medical Center under the Ministry of Health and Welfare was involved in the design and conduct of this research, but it was not possible to involve patients in our research.

## Results

### Study population

**Figure 1:**
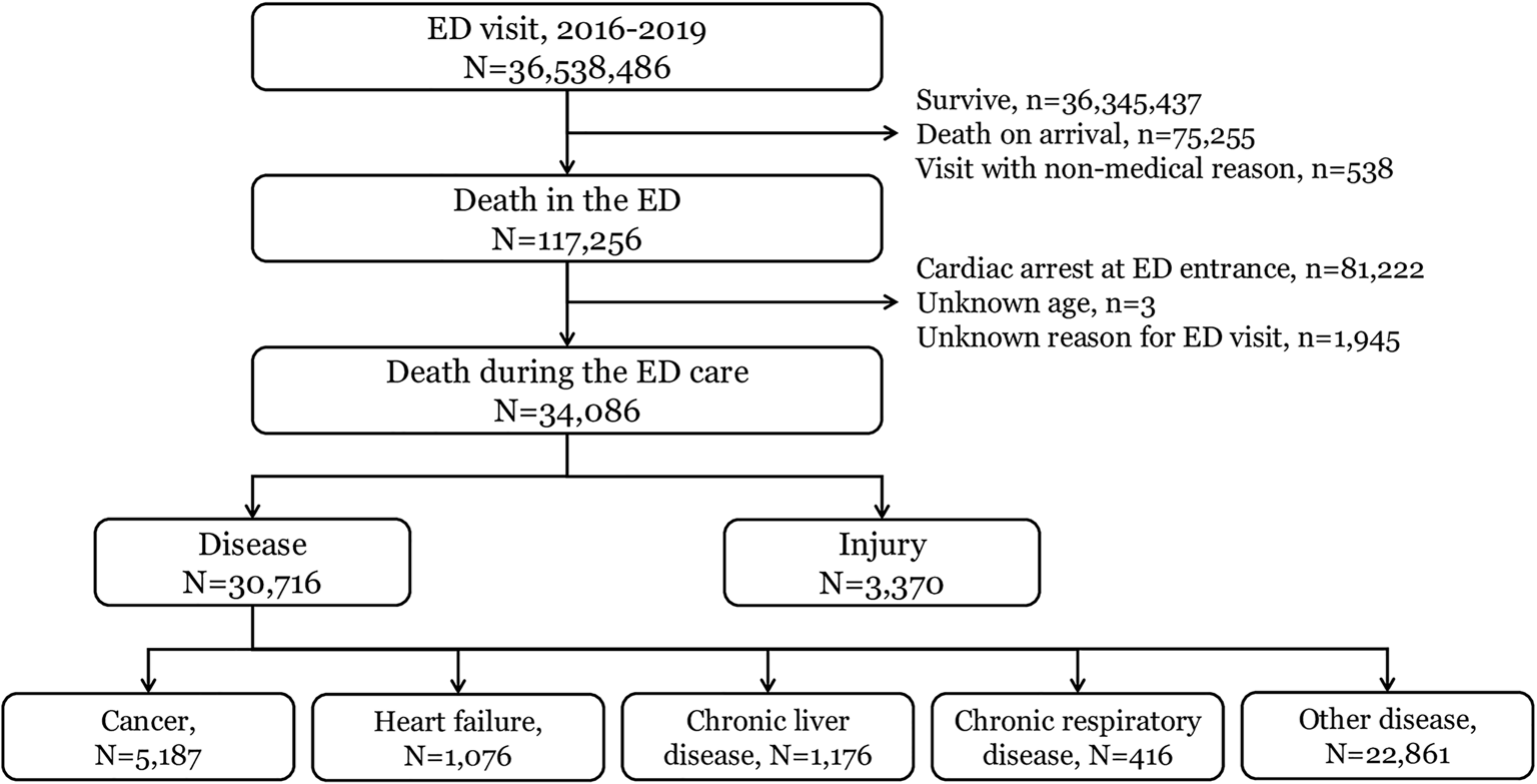
Study population. ED, emergency department.

Between 2016 and 2019, the total number of ED visits was 36,538,486 of which total death toll was 117,256 (0.3%). Among them, 34,086 ED deaths were included as a study population, excluding cases that were already in cardiac arrest at the ED entrance (n = 81,222) and cases with unknown information on age (n = 3) and reason for ED visit (n = 1945) (Fig. 1). Approximately 8500 patients per year died in the ED during the ED care.

### Characteristics of the ED deaths

Among the 34,086 ED deaths, 3370 (9.9%) died from injury while 30,716 (90%) died from diseases. The median age of ED deaths was 75 (63–83) years old. 32.2% (n = 10,961) had an alert mental status upon ED arrival. Median (IQR) ED-LOS was 4.4 (2.0–11.2) h and 32.8% (n = 11,183) stayed in the ED over 8 h before death. CPR was provided in 45.4% and endotracheal intubation was performed in 48.6%. Compared to the injury-related ED deaths group, the disease-related ED deaths group was older (76 (64–83) vs. 68 (54–79) years old, p < 0.01) and had longer ED-LOS (4.5 (1.9–11.7) vs. 3.6 (2.0–6.9) h, p < 0.01) (Table 1).

**Table 1:** Demographics of patients who died in the emergency department in Korea, 2016 to 2019.

	Total		Disease-related		Injury-related		P value
	N	%	N	%	N	%	
Total	34,086	100.0	30,716	100.0	3370	100.0	
Age, year							< 0.001
0 ~ 18	274	0.8	193	0.6	81	2.4	
19 ~ 64	9312	27.3	7907	25.7	1405	41.7	
65 ~ 120	24,500	71.9	22,616	73.6	1884	55.9	
Median, IQR	75 (63–83)		76 (64–83)		68 (54–79)		< 0.001
Sex, female	14,040	41.2	12,976	42.2	1064	31.6	< 0.001
Insurance, Medicaid	4057	11.9	3825	12.5	232	6.9	< 0.001
Year							< 0.001
2016	8466	24.8	7474	24.3	992	29.4	
2017	8537	25.0	7620	24.8	917	27.2	
2018	8711	25.6	7955	25.9	756	22.4	
2019	8372	24.6	7667	25.0	705	20.9	
ED visit time							
Nighttime	13,578	39.8	12,182	39.7	1396	41.4	0.047
Weekend	9803	28.8	8893	29.0	910	27.0	0.018
Use of ambulance	18,715	54.9	16,265	53.0	2450	72.7	< 0.001
Route of ED visit							
Transfer-in	11,440	33.6	10,632	34.6	808	24.0	< 0.001
Metropolitan ED	16,796	49.3	15,442	50.3	1354	40.2	< 0.001
Level of ED							< 0.001
Level 1	12,536	36.8	11,051	36.0	1485	44.1	
Level 2	16,828	49.4	15,278	49.7	1550	46.0	
Level 3	4722	13.9	4387	14.3	335	9.9	
Mental status at ED entrance							< 0.001
Alert	10,961	32.2	10,398	33.9	563	16.7	
Verbal	6486	19.0	5611	18.3	875	26.0	
Pain	7793	22.9	6581	21.4	1212	36.0	
Unresponsiveness	4127	12.1	3745	12.2	382	11.3	
Unknown	4719	13.8	4381	14.3	338	10.0	
Length of stay, h							< 0.001
0 ~ 1	3647	10.7	3409	11.1	238	7.1	
2 ~ 4	12,660	37.1	11,034	35.9	1626	48.2	
5 ~ 8	6592	19.3	5817	18.9	775	23.0	
9 more	11,183	32.8	10,452	34.0	731	21.7	
Median, IQR	4.4 (2.0–11.2)		4.5 (1.9–11.7)		3.6 (2.0–6.9)		< 0.001
Life-sustaining treatment							
CPR	15,466	45.4	13,562	44.2	1904	56.5	< 0.001
Intubation	16,582	48.6	14,333	46.7	2249	66.7	< 0.001

*IQR* interquartile range, *ED* emergency department, *CPR* cardiopulmonary resuscitation.

Among the disease-related ED deaths, the most common diagnoses were cardiac arrest (22.1%), followed by pneumonia (8.6%) and myocardial infarction (4.7%). In cancer patients, the most common diagnoses excluding cancer diagnoses (42.7%) were pneumonia (6.5%), followed by cardiac arrest (6.3%) and dyspnea (3.8%) (Table 2).

**Table 2:** The 15 most frequent diagnoses for disease-related deaths in the emergency department.

Disease-related deaths			Cancer-related deaths		
Diagnoses (ICD-10 code)	N	%	Diagnoses (ICD-10 code)	N	%
Cardiac arrest (I46)	6801	22.1	Cancer diagnosis (C00–C99)	2217	42.7
Pneumonia (J12–J18)	2654	8.6	Pneumonia (J12–J18)	336	6.5
Myocardial infarction (I21–I23)	1455	4.7	Cardiac arrest (I46)	327	6.3
Dyspnea (R06, J96)	1196	3.9	Dyspnea (R06, J96)	199	3.8
Shock (R57, I95)	1119	3.6	Other gastrointestinal problem (K92)	196	3.8
Other gastrointestinal problem (K92)	1043	3.4	Shock (R57, I95)	150	2.9
Brain hemorrhage (I60–62)	935	3.0	Sepsis (A41)	126	2.4
Sepsis (A41)	883	2.9	Other disorders of fluid, electrolyte and acid–base balance (E87)	93	1.8
Lung cancer (C33–C34)	850	2.8	Unknown death (R99)	70	1.3
Liver cancer (C22)	803	2.6	Agranulocytosis (D70)	50	1.0
Unknown death (R99)	789	2.6	Other disorders of pancreatic internal secretion (E16)	46	0.9
Aortic aneurysm and dissection (I71–I72)	651	2.1	Osteoporosis without pathological fracture (M81)	46	0.9
Renal failure (N17–N18)	579	1.9	Liver cirrhosis (K74)	44	0.8
Other disorders of fluid, electrolyte and acid–base balance (E87)	544	1.8	Anemia (D64)	37	0.7
Gastric cancer (C16)	321	1.0	Peritonitis (K65)	36	0.7
Others	10,093	32.9	Others	1214	23.4

### Characteristics of the disease-related ED deaths

Among 30,716 disease-related ED deaths, 7855 (25.6%) were ED deaths of patients with palliative care-eligible: 16.9% (n = 5187) was related with cancer, 1.4% (n = 416) with chronic respiratory disease, 3.8% (n = 1176) with chronic liver disease, and 3.5% (n = 1076) with heart failure. The ED deaths of patients with palliative care-eligible conditions had longer median ED-LOS, compared to ED deaths of patients without palliative care-eligible conditions (p < 0.01). Regarding life-sustaining treatment, 2327/7855 (29.6%) received CPR and 2707/7855 (34.5%) received endotracheal intubation before death. Cancer patients received relatively less life-sustaining treatment: 23.4% received CPR and 27.1% received endotracheal intubation (Table 3). Demographics according to the ED-LOS are presented in Supplementary Table S1.

**Table 3:** Demographics of disease-related deaths in the emergency department according to the palliative care-eligible disease.

	Total		Cancer		Chronic respiratory disease		Chronic liver disease		Heart failure		Others		P-value
	N	%	N	%	N	%	N	%	N	%	N	%	
Total	30,716	100.0	5187	100.0	416	100.0	1176	100.0	1076	100.0	22,861	100.0	
Age, year													< 0.001
0 ~ 18	193	0.6	9	0.2	2	0.5	0	0.0	5	0.5	177	0.8	
19 ~ 64	7907	25.7	1812	34.9	49	11.8	784	66.7	103	9.6	5159	22.6	
65 ~ 120	22,616	73.6	3366	64.9	365	87.7	392	33.3	968	90.0	17,525	76.7	
Median, IQR	76 (64–83)		70 (60–78)		79 (72–84)		58 (51–69)		82 (75–87)		77 (66–84)		< 0.001
Sex, female	12,976	42.2	1784	34.4	119	28.6	280	23.8	637	59.2	10,156	44.4	< 0.001
Insurance, medicaid	3825	12.5	464	8.9	53	12.7	229	19.5	159	14.8	2920	12.8	< 0.001
Year													0.005
2016	7474	24.3	1190	22.9	106	25.5	293	24.9	243	22.6	5642	24.7	
2017	7620	24.8	1272	24.5	106	25.5	282	24.0	297	27.6	5663	24.8	
2018	7955	25.9	1317	25.4	115	27.6	326	27.7	281	26.1	5916	25.9	
2019	7667	25.0	1408	27.1	89	21.4	275	23.4	255	23.7	5640	24.7	
ED visit time													
Nighttime	12,182	39.7	2094	40.4	161	38.7	513	43.6	412	38.3	9002	39.4	0.032
Weekend	8893	29.0	1432	27.6	121	29.1	346	29.4	306	28.4	6688	29.3	0.211
Use of ambulance	16,265	53.0	2687	51.8	208	50.0	636	54.1	495	46.0	12,239	53.5	< 0.001
Route of ED visit													
Transfer-in	10,632	34.6	1579	30.4	152	36.5	403	34.3	424	39.4	8074	35.3	< 0.001
Metropolitan	15,442	50.3	2944	56.8	178	42.8	651	55.4	464	43.1	11,205	49.0	
Level of ED													< 0.001
Level 1	11,051	36.0	1796	34.6	148	35.6	487	41.4	393	36.5	8227	36.0	
Level 2	15,278	49.7	2935	56.6	204	49.0	595	50.6	501	46.6	11,043	48.3	
Level 3	4387	14.3	456	8.8	64	15.4	94	8.0	182	16.9	3591	15.7	
Mental status at ED entrance													< 0.001
Alert	10,398	33.9	2515	48.5	188	45.2	507	43.1	506	47.0	6682	29.2	
Verbal response	5611	18.3	871	16.8	59	14.2	227	19.3	154	14.3	4300	18.8	
Pain response	6581	21.4	583	11.2	52	12.5	147	12.5	103	9.6	5696	24.9	
Unresponsiveness	3745	12.2	758	14.6	52	12.5	199	16.9	133	12.4	2603	11.4	
Unknown	4381	14.3	460	8.9	65	15.6	96	8.2	180	16.7	3580	15.7	
Length of stay, h													< 0.001
0 ~ 1	3409	11.1	353	6.8	27	6.5	34	2.9	63	5.9	2932	12.8	
2 ~ 4	11,034	35.9	1272	24.5	127	30.5	231	19.6	419	38.9	8985	39.3	
5 ~ 8	5817	18.9	1136	21.9	92	22.1	229	19.5	240	22.3	4120	18.0	
9 ~	10,452	34.0	2426	46.8	170	40.9	682	58.0	354	32.9	6820	29.8	
Median, IQR	4.5 (1.9–11.7)		7.3 (3.2–15.9)		6.1 (2.7–16.5)		10.1 (4.6–20.0)		4.6 (2.4–10.8)		3.8 (1.7–10.0)		< 0.001
Life-sustaining treatment													
CPR	13,562	44.2	1216	23.4	143	34.4	487	41.4	481	44.7	11,235	49.1	< 0.001
Intubation	14,333	46.7	1407	27.1	169	40.6	613	52.1	518	48.1	11,626	50.9	< 0.001

*IQR* interquartile range, *ED* emergency department, *CPR* cardiopulmonary resuscitation.

Regarding provision of CPR, 55.8% (n = 17,154) of the 30,716 disease-related ED deaths did not receive CPR prior to death. Patients who did not receive CPR had longer ED-LOS than those who received CPR (6.5 (2.6–16.0) vs. 3.0 (1.6–6.8) h, p < 0.01). Of the ED deaths that received CPR, 17.2% were diagnosed with palliative care-eligible diseases (cancer 9.0%, chronic respiratory disease 1.1%, chronic liver disease 3.6%, and heart failure 3.5%). (Table 4). The provision of CPR for the ED deaths of patients with palliative care-eligible diseases are presented in Supplement Table S2.

**Table 4:** Demographics of disease-related deaths in the emergency department according to the provision of cardiopulmonary resuscitation.

	Total		No CPR		CPR		P-value
	N	%	N	%	N	%	
Total	30,716	100.0	17,154	100.0	13,562	100.0	
Age, year							< 0.001
0 ~ 18	193	0.6	58	0.3	135	1.0	
19 ~ 64	7907	25.7	3772	22.0	4135	30.5	
65 ~ 120	22,616	73.6	13,324	77.7	9292	68.5	
Median, IQR	76 (64–83)		78 (66–85)		74 (61–81)		< 0.001
Sex, female	12,976	42.2	7534	43.9	5442	40.1	< 0.001
Insurance, medicaid	3825	12.5	2202	12.8	1623	12.0	< 0.001
Year							0.005
2016	7474	24.3	4145	24.2	3329	24.5	
2017	7620	24.8	4157	24.2	3463	25.5	
2018	7955	25.9	4454	26.0	3501	25.8	
2019	7667	25.0	4398	25.6	3269	24.1	
ED visit time							
Nighttime	12,182	39.7	6824	39.8	5358	39.5	0.627
Weekend	8893	29.0	5004	29.2	3889	28.7	0.342
Use of ambulance	16,265	53.0	8040	46.9	8225	60.6	< 0.001
Route of ED visit							
Transfer-in	10,632	34.6	6725	39.2	3907	28.8	< 0.001
Metropolitan	15,442	50.3	8618	50.2	6824	50.3	0.892
Level of ED							0.022
Level 1	11,051	36.0	6123	35.7	4928	36.3	
Level 2	15,278	49.7	8643	50.4	6635	48.9	
Level 3	4387	14.3	2388	13.9	1999	14.7	
Mental status at ED entrance							< 0.001
Alert	10,398	33.9	5959	34.7	4439	32.7	
Verbal response	5611	18.3	3682	21.5	1929	14.2	
Pain response	6581	21.4	2757	16.1	3824	28.2	
Unresponsiveness	3745	12.2	2391	13.9	1354	10.0	
Unknown	4381	14.3	2365	13.8	2016	14.9	
Length of stay, h							< 0.001
0 ~ 1	3409	11.1	1717	10.0	1692	12.5	
2 ~ 4	11,034	35.9	4507	26.3	6527	48.1	
5 ~ 8	5817	18.9	3353	19.5	2464	18.2	
9 ~	10,452	34.0	7576	44.2	2876	21.2	
Median, IQR	4.5 (1.9–11.7)		6.5 (2.6–16)		3.0 (1.6–6.8)		< 0.001
Palliative care eligible disease							< 0.001
Cancer	5187	16.9	3971	23.1	1216	9.0	
Chronic respiratory disease	416	1.4	273	1.6	143	1.1	
Chronic liver disease	1176	3.8	689	4.0	487	3.6	
Heart failure	1076	3.5	595	3.5	481	3.5	
Life-sustaining treatment							
Intubation	14,333	46.7	3420	19.9	10,913	80.5	< 0.001

*CPR* cardiopulmonary resuscitation, *IQR* interquartile range, *ED* emergency department.

### Epidemiologic trends of ED deaths

**Figure 2:**
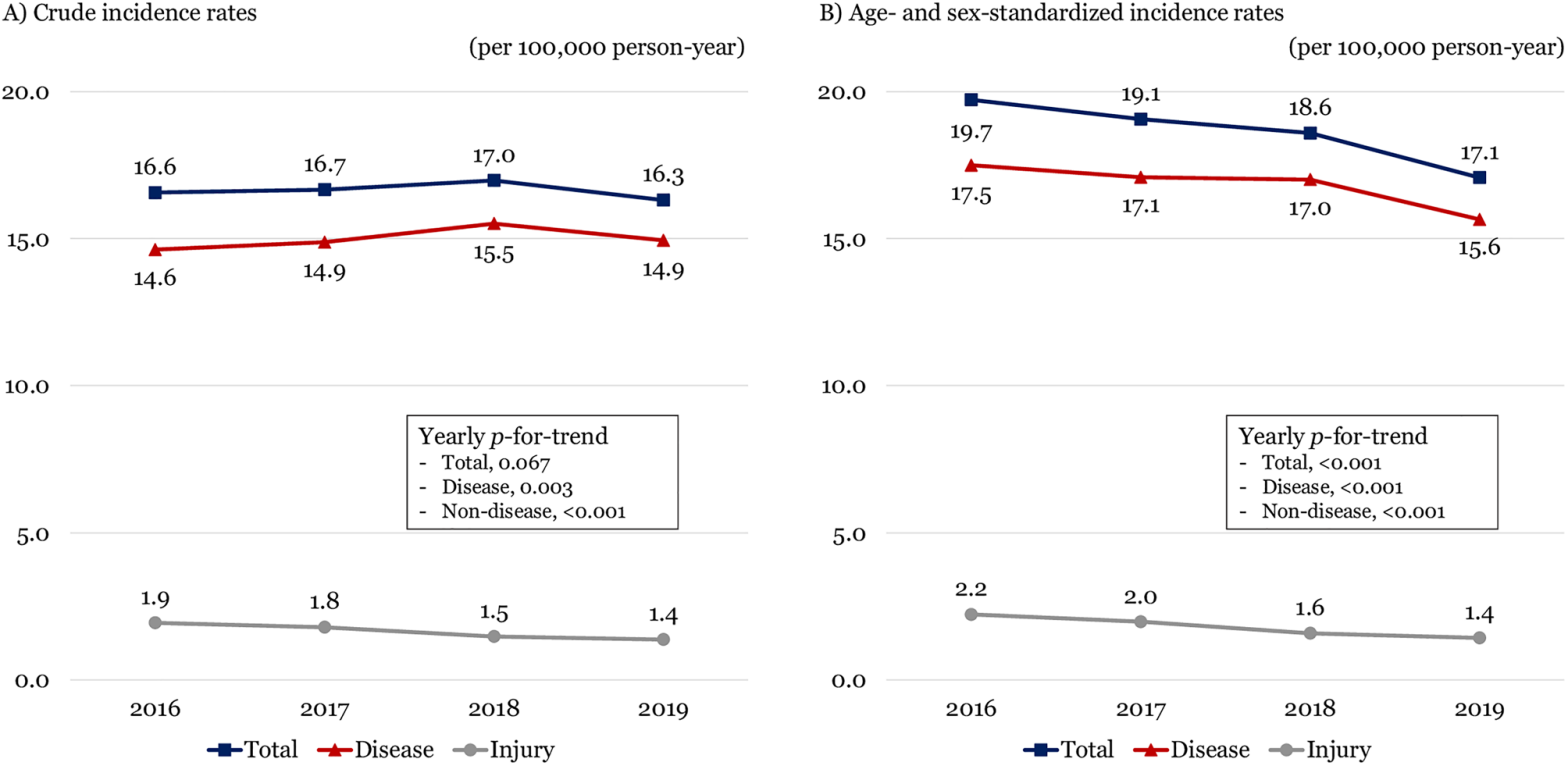
Trends of the crude and age- and sex- standardized incidence rates per 100,000 person-year of emergency department deaths from 2016 to 2019. (**A**) Crude rate. (**B**) Age- and sex-standardized rate.

**Figure 3:**
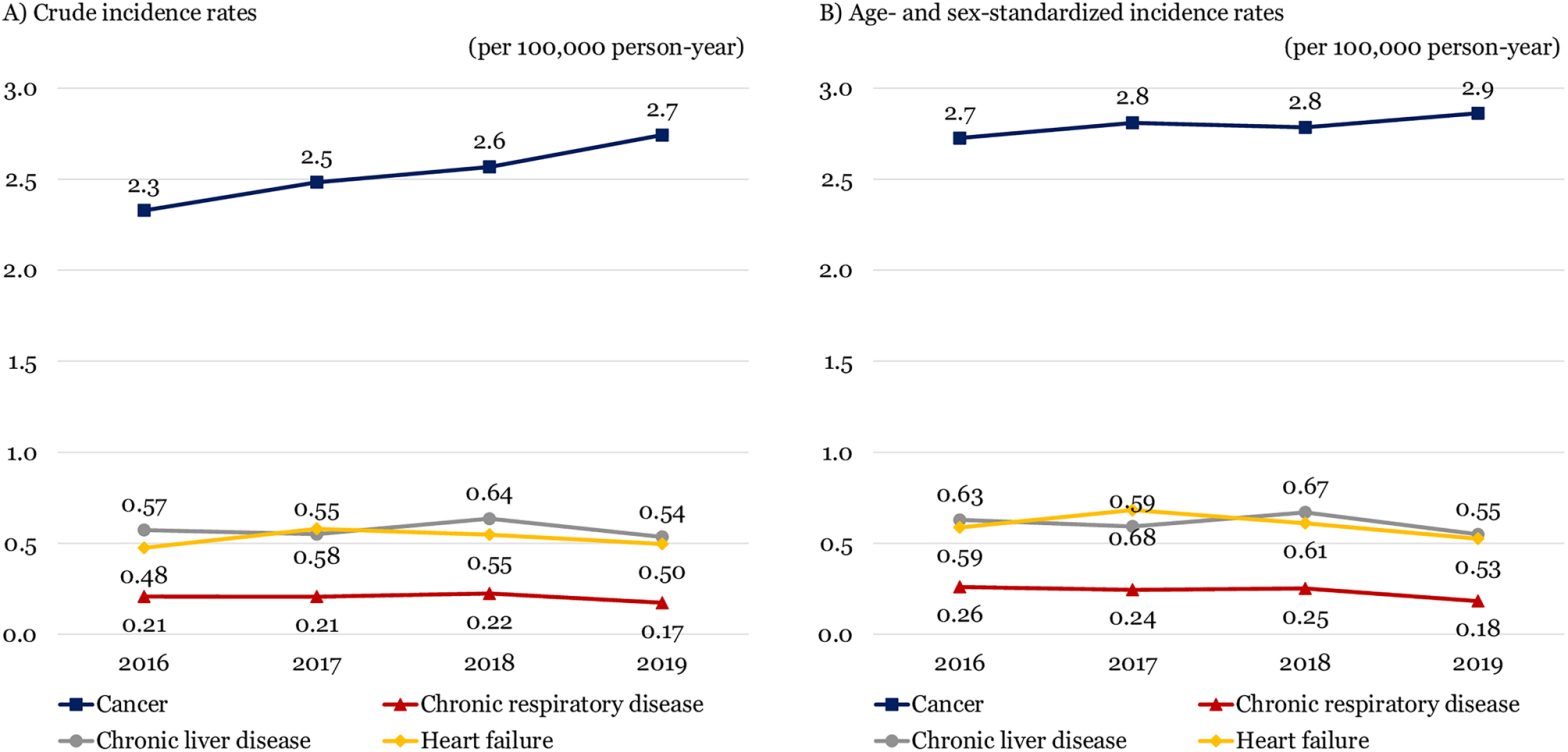
Trends of the crude and age- and sex- standardized incidence rates per 100,000 person-year of emergency department deaths with palliative care-eligible disease from 2016 to 2019. (**A**) Crude rate. (**B**) Age- and sex-standardized rate.

The crude incidence rate of ED deaths per 100,000 person-years was 16.6 in 2016, 16.7 in 2017, 17.0 in 2018, and 16.3 in 2019, respectively, showing a steady trend during the study period (p-for-trend = 0.067). The age- and sex- standardized incidence rate of ED deaths per 100,000 person-years decreased from 19.7 in 2016 to 17.1 in 2019 (p-for-trend < 0.001). Both disease (17.5 to 15.6) and injury (2.2 to 1.4) were on the decline (p-for-trend < 0.001) (Fig. 2). ED deaths with cancer were slightly increased with crude rate from 2.3 to 2.7 and standardized rate from 2.7 to 2.9 (Fig. 3).

## Discussion

During the 4 years between 2016 and 2019, a total of 34,086 patients died in the ED during care, approximating to 2.9% of all deaths in Korea. Among those ED deaths, 90% were disease-related death. Patients stayed for a median 4.4 h before death in the ED, of which only 45.4% received CPR. A quarter of disease-related ED deaths had a condition eligible for palliative care. They stayed in the ED longer while receiving less life-sustaining treatment (29.6% received CPR) before death, compared to other patients. Cancer-related ED deaths accounted for 16% of disease-related ED deaths: half of them died after staying for more than 8 h in the ED and a quarter of them received CPR before death. This study found that nearly 8500 patients die in the ED every year and the annual incidence rate of ED deaths was about 17 per 100,000 person-year in Korea. To the best of current knowledge, it is the first study to investigate the magnitude of ED deaths nationwide.

In Korea, the death rates per 100,000 person-years in 2019 were 574.8 for all cause of death and 158.2 due to cancer, accounting for 27.5% of all deaths^27^. Since the ED is for providing acute care for critically ill patients, death in the ED is unavoidable. However, not much is known about patients who die in an ED^13,34^. In this study, only 10% of the ED deaths were caused by injuries such as traffic accidents or falls. This was smaller than a single center study done in Switzerland^10^. 90% of the ED deaths were disease-related patients, and one quarter of them had diagnosis codes subject to palliative care. Previous studies have reported that 30 ~ 50% of death in the ED were related to serious chronic illnesses or palliative care needs^16,17,35^. Since this study classified only four representative diseases as palliative care-eligible diseases, size of the palliative care subjects may be smaller than other studies. Theoretically, all patients with imminent death are eligible for palliative care, including those who die in the ED^2^.

In this study, ED deaths happened after a median 4.4 h in the ED, and 30% of disease-related ED deaths occurred after 8 h in the ED. It was previously reported in US that all ED visit patients stayed for median 4 h in the ED, and that more than 8 h of stay is defined as a long stay^31,32^. Other studies reported that, among ED visits, discharged patients stayed for median 2.5 h and hospitalized patients stayed for 4 h^36,37^. Patients who die in ED may stay for relatively longer periods of time in the ED and utilize more resources than patients who survived. Among all ED deaths, about half of ED deaths received CPR (45.4%) and endotracheal intubation (48.6%) before death in this study. It aligns with other studies in that more than 50% of the patients died in the ED without CPR^38–40^. Moreover, 30% of ED deaths with palliative care-eligible diseases and a quarter of cancer-related ED deaths received CPR before death, which is 4–7 times higher than that of a German study^41^. CPR is a life-saving treatment for acute stage patients, but it could be a life-sustaining treatment with little meaning for incurable and terminally ill patients. A hospital-level study or qualitative study is needed to know how life-sustaining treatment is provided in individual patients. The results of this study show the possibility that there still is a substantial unmet need for palliative care in the ED and the need for follow-up studies^42,43^.

In this study, about half of ED deaths with palliative care-eligible diseases and cancer-related ED deaths stayed over 8 h before death in the ED. During ED care, 30% of ED deaths with palliative care-eligible diseases and a quarter of cancer-related ED deaths received CPR before death. More attention would be needed on how to provide end-of-life care in times of expected death in the ED. This discussion encompasses not only which care should be provided for dying patients, but also whether it would be suitable for such patients to stay in ED for such long times. This study showed overall epidemiologic characteristics of ED deaths using a nationally representative emergency database. Considering that patients who died within 72 h after visiting the ED, as well as ED death, are also eligible for palliative care, the need for palliative care at the end-of-life in the ED would increase (Supplement Table S3). Based on the need for palliative care at end-of-life in the ED in this study, it is necessary to develop a strategy for improving end-of-life care in the ED.

This study has several limitations. First, the NEDIS database does not collect information on whether the patients have previously completed advance directives or physician orders for life-sustaining treatment (POLST). Further study is needed to determine whether patients with palliative-care eligible conditions have a plan before visiting the ED and can receive proper end-of-life care through this. As a limitation of the studies using a large database, it is necessary to comprehensively interpret the results of this study by referring to the results of studies focusing on individual patients. Second, whether the patient had a disease subject to palliative care was determined based on the diagnosis codes in the ED medical records. Whether that disease was previously diagnosed or first diagnosed in the ED is unknown. Third, the NEDIS data does not collect information of test results and medication prescription. It is difficult to know whether the dying patient received sufficient supportive care in the ED before death, such as pain control. Fourth, Korea has the NHI service is implemented, and palliative care services are in the introduction stage. It is difficult to generalize the result to other countries with different healthcare system and accessibility to palliative care.

In conclusion, more than 30,000 people died during care in the ED over the past four years. At least a quarter of disease-related ED deaths were owed to palliative care-eligible diseases. More than 30% of disease-related ED deaths and 40% of cancer-related ED deaths happened at least 8 h after arrival in the ED. About half of disease-related ED deaths and 30% of ED deaths with palliative care-eligible diseases received CPR before death. Increased efforts to understand death in the ED may improve end-of-life care in the ED.

## Supplementary Information

Supplementary Tables.

## Data Availability

The data of this study were obtained from the National Emergency Medical Center under the Ministry of Health and Welfare in Korea but restrictions apply to the availability of these data and so are not publicly available, but are available from the corresponding author on reasonable request.
