# Publisher Correction: Epidemiology of patients who died in the emergency departments and need of end-of-life care in Korea from 2016 to 2019

**DOI:** 10.1038/s41598-023-29430-1

**Published:** 2023-02-13

**Authors:** Sun Young Lee, Young Sun Ro, Sang Do Shin, Eunsil Ko, Seong Jung Kim

**Affiliations:** 1 Public Healthcare Center, Seoul National University Hospital, Seoul, Korea; 2 Laboratory of Emergency Medical Services, Seoul National University Hospital Biomedical Research Institute, Seoul, Korea; 3 Department of Medicine, Seoul National University College of Medicine, Seoul, Korea; 4 Department of Emergency Medicine, Seoul National University Hospital, 101 Daehak-ro, Jongno-gu, Seoul, 03080 Korea; 5 Department of Emergency Medicine, Seoul National University College of Medicine, Seoul, Korea; 6 National Emergency Medical Center, National Medical Center, Seoul, Korea; 7 Department of Emergency Medicine, Chosun University Hospital, 365 Pilmun-daero, Dong-gu, Gwangju, 61453 Korea

Correction to: *Scientific Reports* 10.1038/s41598-023-27947-z, published online 13 January 2023.

The original version of this Article contained errors in Tables 1 and 3.

In Table 1, the “Median, IQR” of the age of “Total”, “Disease-related” and “Injury-related” patients as well as the “P-value” were shifted. Furthermore, the “Median, IQR” of these patients’ “Length of stay” and the “P-value” were shifted.

Incorrect:

**Table:** 

	Total		Disease-related		Injury-related		P value
	N	%	N	%	N	%	
Median, IQR	75 (63–83)	76 (64–83)	68 (54–79)	< 0.001			
Median, IQR	4.4 (2.0–11.2)	4.5 (1.9–11.7)	3.6 (2.0–6.9)	< 0.001			

Correct:

**Table:** 

	Total		Disease-related		Injury-related		P value
	N	%	N	%	N	%	
Median, IQR	75 (63–83)		76 (64–83)		68 (54–79)		< 0.001
Median, IQR	4.4 (2.0–11.2)		4.5 (1.9–11.7)		3.6 (2.0–6.9)		< 0.001

Additionally, in Table 3, the “P-value” for “Mental status at ED entrance” was shifted.

Incorrect:

**Table:** 

	Total		Cancer		Chronic respiratory disease		Chronic liver disease		Heart failure		Others		P-value
	N	%	N	%	N	%	N	%	N	%	N	%	
Mental status at ED entrance												< 0.001	

Correct:

**Table:** 

	Total		Cancer		Chronic respiratory disease		Chronic liver disease		Heart failure		Others		P-value
	N	%	N	%	N	%	N	%	N	%	N	%	
Mental status at ED entrance													< 0.001

The original Article has been corrected.

