# Supplementary material for: Epidemiology of patients who died in the emergency departments and need of end-of-life care in Korea from 2016 to 2019
Source: Sci Rep. 2023 Jan 13;13:686. doi: 10.1038/s41598-023-27947-z (PMC9839758; doi:10.1038/s41598-023-27947-z)
Supplement: Supplementary file 1 — Supplementary Tables. [file 41598_2023_27947_MOESM1_ESM.docx]

Supplement table 1. Demographics of disease-related deaths in the emergency department according to the length of stay*

|  |  | 0~1 hours | | | 2~4 hours | | | | 5~8 hours | | | | Over 8 hours | | | | P- value |
| --- | --- | --- | --- | --- | --- | --- | --- | --- | --- | --- | --- | --- | --- | --- | --- | --- | --- |
|  |  | N | % | | N | | % | | N | | % | | N | | % | |  |
| Total | | 3409 | 100.0 | | 11034 | | 100.0 | | 5817 | | 100.0 | | 10452 | | 100.0 | |  |
| Age, year | |  |  | |  | |  | |  | |  | |  | |  | | <001 |
|  | 0~18 | 35 | 1.0 | | 91 | | 0.8 | | 41 | | 0.7 | | 26 | | 0.2 | |  |
|  | 19~64 | 809 | 23.7 | | 2679 | | 24.3 | | 1473 | | 25.3 | | 2945 | | 28.2 | |  |
|  | 65~120 | 2565 | 75.2 | | 8264 | | 74.9 | | 4303 | | 74.0 | | 7481 | | 71.6 | |  |
|  | Median, IQR | 78 (65–85) | | | 76 (64–83) | | | | 76 (64–83) | | | | 75 (62–82) | | | | <0.01 |
| Sex, female | | 1502 | 44.1 | | 4765 | | 43.2 | | 2463 | | 42.3 | | 4246 | | 40.6 | | <0.01 |
| Insurance, Medicaid | | 353 | 10.4 | | 1280 | | 11.6 | | 741 | | 12.7 | | 1450 | | 13.9 | | <0.01 |
| Year | |  |  | |  | |  | |  | |  | |  | |  | | <0.01 |
|  | 2016 | 910 | 26.7 | | 2682 | | 24.3 | | 1358 | | 23.3 | | 2524 | | 24.1 | |  |
|  | 2017 | 978 | 28.7 | | 2887 | | 26.2 | | 1397 | | 24.0 | | 2358 | | 22.6 | |  |
|  | 2018 | 813 | 23.8 | | 2738 | | 24.8 | | 1505 | | 25.9 | | 2898 | | 27.7 | |  |
|  | 2019 | 708 | 20.8 | | 2727 | | 24.7 | | 1557 | | 26.8 | | 2672 | | 25.6 | |  |
| ED visit time | |  |  | |  | |  | |  | |  | |  | |  | |  |
|  | Nighttime | 1299 | 38.1 | | 4372 | | 39.6 | | 2308 | | 39.7 | | 4201 | | 40.2 | | 0.30 |
|  | Weekend | 1028 | 30.2 | | 3160 | | 28.6 | | 1674 | | 28.8 | | 3030 | | 29.0 | | 0.55 |
| Use of ambulance | | 2146 | 63.0 | | 6683 | | 60.6 | | 2946 | | 50.6 | | 4488 | | 42.9 | | <0.01 |
| Route of ED visit | |  |  | |  | |  | |  | |  | |  | |  | |  |
|  | Transfer-in | 460 | 13.5 | | 3135 | | 28.4 | | 2276 | | 39.1 | | 4759 | | 45.5 | | <0.01 |
| Metropolitan | | 1188 | 34.8 | | 5249 | | 47.6 | | 3034 | | 52.2 | | 5970 | | 57.1 | |  |
| Level of ED | |  |  | |  | |  | |  | |  | |  | |  | | <0.01 |
|  | Level 1 | 519 | 15.2 | | 3474 | | 31.5 | | 2307 | | 39.7 | | 4751 | | 45.5 | |  |
|  | Level 2 | 1240 | 36.4 | | 5453 | | 49.4 | | 3034 | | 52.2 | | 5548 | | 53.1 | |  |
|  | Level 3 | 1650 | 48.4 | | 2107 | | 19.1 | | 476 | | 8.2 | | 153 | | 1.5 | |  |
| Mental status at ED entrance | | | |  | |  | |  | |  | |  | |  | |  | <0.01 |
|  | Alert | 140 | 4.1 | | 2691 | | 24.4 | | 2339 | | 40.2 | | 5226 | | 50.0 | |  |
|  | Verbal response | 271 | 7.9 | | 2105 | | 19.1 | | 1182 | | 20.3 | | 2053 | | 19.6 | |  |
|  | Pain response | 1258 | 36.9 | | 2955 | | 26.8 | | 1005 | | 17.3 | | 1362 | | 13.0 | |  |
|  | Unresponsiveness | 85 | 2.5 | | 1192 | | 10.8 | | 815 | | 14.0 | | 1653 | | 15.8 | |  |
|  | Unknown | 1655 | 48.5 | | 2091 | | 19.0 | | 476 | | 8.2 | | 158 | | 1.5 | |  |
| Palliative care eligible disease | | | |  | |  | |  | |  | |  | |  | |  | <0.01 |
|  | Cancer | 353 | 10.4 | | 1272 | | 11.5 | | 1136 | | 19.5 | | 2426 | | 23.2 | |  |
|  | Chronic respiratory disease | 27 | 0.8 | | 127 | | 1.2 | | 92 | | 1.6 | | 170 | | 1.6 | |  |
|  | Chronic liver disease | 34 | 1.0 | | 231 | | 2.1 | | 229 | | 3.9 | | 682 | | 6.5 | |  |
|  | Heart failure | 63 | 1.8 | | 419 | | 3.8 | | 240 | | 4.1 | | 354 | | 3.4 | |  |
| Life-sustaining treatment | | | |  | |  | |  | |  | |  | |  | |  |  |
|  | CPR | 1692 | 49.6 | | 6527 | | 59.2 | | 2464 | | 42.4 | | 2876 | | 27.5 | | <0.01 |
|  | Intubation | 963 | 28.2 | | 4649 | | 42.1 | | 2602 | | 44.7 | | 4445 | | 42.5 | | <0.01 |

IQR, interquartile range; ED, emergency department; CPR, cardiopulmonary resuscitation

*Four patients with unknown length of stay in the ED were excluded.

Supplement table 2. Demographics of emergency department deaths from palliative care-eligible diseases according to the provision of cardiopulmonary resuscitation

|  |  | Total | | No CPR | | CPR | | P-value |
| --- | --- | --- | --- | --- | --- | --- | --- | --- |
|  |  | N | % | N | % | N | % |  |
| Total | | 7855 | 100.0 | 5528 | 100.0 | 2327 | 100.0 |  |
| Age, year | |  |  |  |  |  |  | <0.01 |
|  | 0~18 | 16 | 0.2 | 8 | 0.1 | 8 | 0.3 |  |
|  | 19~64 | 2748 | 35.0 | 1857 | 33.6 | 891 | 38.3 |  |
|  | 65~120 | 5091 | 64.8 | 3663 | 66.3 | 1428 | 61.4 |  |
|  | Median, IQR | 71 (59–80) | | 72 (60–80) | | 70 (58–79) | | <0.01 |
| Sex, female | | 2820 | 35.9 | 1998 | 36.1 | 822 | 35.3 | 0.49 |
| Insurance, Medicaid | | 905 | 11.5 | 630 | 11.4 | 275 | 11.8 | 0.39 |
| Year | |  |  |  |  |  |  | 0.84 |
|  | 2016 | 1832 | 23.3 | 1303 | 23.6 | 529 | 22.7 |  |
|  | 2017 | 1957 | 24.9 | 1375 | 24.9 | 582 | 25.0 |  |
|  | 2018 | 2039 | 26.0 | 1423 | 25.7 | 616 | 26.5 |  |
|  | 2019 | 2027 | 25.8 | 1427 | 25.8 | 600 | 25.8 |  |
| ED visit time | |  |  |  |  |  |  |  |
|  | Nighttime | 3180 | 40.5 | 2252 | 40.7 | 928 | 39.9 | 0.48 |
|  | Weekend | 2205 | 28.1 | 1583 | 28.6 | 622 | 26.7 | 0.09 |
| Use of ambulance | | 4026 | 51.3 | 2770 | 50.1 | 1256 | 54.0 | <0.01 |
| Route of ED visit | |  |  |  |  |  |  |  |
|  | Transfer-in | 2558 | 32.6 | 1875 | 33.9 | 683 | 29.4 | <0.01 |
| Metropolitan | | 4237 | 53.9 | 3032 | 54.8 | 1205 | 51.8 | 0.01 |
| Level of ED | |  |  |  |  |  |  | <0.01 |
|  | Level 1 | 2824 | 36.0 | 1896 | 34.3 | 928 | 39.9 |  |
|  | Level 2 | 4235 | 53.9 | 3051 | 55.2 | 1184 | 50.9 |  |
|  | Level 3 | 796 | 10.1 | 581 | 10.5 | 215 | 9.2 |  |
| Mental status at ED entrance | |  |  |  |  |  |  | <0.01 |
|  | Alert | 3716 | 47.3 | 2516 | 45.5 | 1200 | 51.6 |  |
|  | Verbal response | 1311 | 16.7 | 1016 | 18.4 | 295 | 12.7 |  |
|  | Pain response | 885 | 11.3 | 560 | 10.1 | 325 | 14.0 |  |
|  | Unresponsiveness | 1142 | 14.5 | 853 | 15.4 | 289 | 12.4 |  |
|  | Unknown | 801 | 10.2 | 583 | 10.5 | 218 | 9.4 |  |
| Length of Stay, hour | |  |  |  |  |  |  | <0.01 |
|  | 0~1 | 477 | 6.1 | 339 | 6.1 | 138 | 5.9 |  |
|  | 2~4 | 2049 | 26.1 | 1230 | 22.3 | 819 | 35.2 |  |
|  | 5~8 | 1697 | 21.6 | 1137 | 20.6 | 560 | 24.1 |  |
|  | 9~ | 3632 | 46.2 | 2822 | 51.0 | 810 | 34.8 |  |
|  | Median, IQR | 7.1 (3.1–15.9) | | 8.3 (3.5–18.4) | | 5.1 (2.5–11.1) | | <0.01 |
| Palliative care eligible disease | |  |  |  |  |  |  | <0.01 |
|  | Cancer | 5187 | 66.0 | 3971 | 71.8 | 1216 | 52.3 |  |
|  | Chronic respiratory disease | 416 | 5.3 | 273 | 4.9 | 143 | 6.1 |  |
|  | Chronic liver disease | 1176 | 15.0 | 689 | 12.5 | 487 | 20.9 |  |
|  | Heart failure | 1076 | 13.7 | 595 | 10.8 | 481 | 20.7 |  |
| Life-sustaining treatment | |  |  |  |  |  |  |  |
|  | Intubation | 2707 | 34.5 | 848 | 15.3 | 1859 | 79.9 | <0.01 |
|  | Mechanical ventilator | 2524 | 32.1 | 942 | 17.0 | 1582 | 68.0 | <0.01 |

CPR, cardiopulmonary resuscitation; IQR, interquartile range; ED, emergency department

Supplement table 3. Demographics of disease-related deaths within 72 hours of visiting the emergency department according to the palliative care-eligible disease

|  |  | Total | | | | Cancer | | | | Chronic respiratory disease | | | Chronic liver disease | | | Heart failure | | | | Others | | | | P-value |
| --- | --- | --- | --- | --- | --- | --- | --- | --- | --- | --- | --- | --- | --- | --- | --- | --- | --- | --- | --- | --- | --- | --- | --- | --- |
|  |  | N | | % | | N | | | % | N | | % | N | | % | N | | % | | N | | % | |  |
| Total | | 95509 | | 100.0 | | 21618 | | | 100.0 | 2480 | | 100.0 | 5579 | | 100.0 | 5685 | | 100.0 | | 60147 | | 100.0 | |  |
| Age, year | |  | |  | |  | | |  |  | |  |  | |  |  | |  | |  | |  | | <0.001 |
|  | 0~18 | 588 | | 0.6 | | 58 | | | 0.3 | 5 | | 0.2 | 10 | | 0.2 | 22 | | 0.4 | | 493 | | 0.8 | |  |
|  | 19~64 | 24585 | | 25.7 | | 7363 | | | 34.1 | 217 | | 8.8 | 3614 | | 64.8 | 516 | | 9.1 | | 12875 | | 21.4 | |  |
|  | 65~120 | 70336 | | 73.6 | | 14197 | | | 65.7 | 2258 | | 91.0 | 1955 | | 35.0 | 5147 | | 90.5 | | 46779 | | 77.8 | |  |
|  | Median, IQR | 76 (64-83) | | | | 71 (60-79) | | | | 80 (73-85) | | | 59 (51-71) | | | 82 (75-87) | | | | 78 (67-84) | | | | <0.001 |
| Sex, female | | 41397 | | 43.3 | | 7480 | | | 34.6 | 834 | | 33.6 | 1422 | | 25.5 | 3470 | | 61.0 | | 28191 | | 46.9 | | <0.001 |
| Insurance, Medicaid | | 12032 | | 12.6 | | 1977 | | | 9.1 | 325 | | 13.1 | 1057 | | 18.9 | 788 | | 13.9 | | 7885 | | 13.1 | | <0.001 |
| Year | |  | |  | |  | | |  |  | |  |  | |  |  | |  | |  | |  | | <0.001 |
|  | 2016 | 22578 | | 23.6 | | 5124 | | | 23.7 | 631 | | 25.4 | 1328 | | 23.8 | 1145 | | 20.1 | | 14350 | | 23.9 | |  |
|  | 2017 | 23650 | | 24.8 | | 5316 | | | 24.6 | 623 | | 25.1 | 1403 | | 25.1 | 1445 | | 25.4 | | 14863 | | 24.7 | |  |
|  | 2018 | 24564 | | 25.7 | | 5389 | | | 24.9 | 647 | | 26.1 | 1431 | | 25.6 | 1526 | | 26.8 | | 15571 | | 25.9 | |  |
|  | 2019 | 24717 | | 25.9 | | 5789 | | | 26.8 | 579 | | 23.3 | 1417 | | 25.4 | 1569 | | 27.6 | | 15363 | | 25.5 | |  |
| ED visit time | |  | |  | |  | | |  |  | |  |  | |  |  | |  | |  | |  | |  |
|  | Nighttime | 34805 | | 36.4 | | 7715 | | | 35.7 | 831 | | 33.5 | 2205 | | 39.5 | 1940 | | 34.1 | | 22114 | | 36.8 | | <0.001 |
|  | Weekend | 26595 | | 27.8 | | 5996 | | | 27.7 | 649 | | 26.2 | 1537 | | 27.5 | 1516 | | 26.7 | | 16897 | | 28.1 | | 0.048 |
| Use of ambulance | | 47652 | | 49.9 | | 10346 | | | 47.9 | 1194 | | 48.1 | 3005 | | 53.9 | 2495 | | 43.9 | | 30612 | | 50.9 | | <0.001 |
| Route of ED visit | |  | |  | |  | | |  |  | |  |  | |  |  | |  | |  | |  | | <0.001 |
|  | Transfer-in | 33818 | | 35.4 | | 6596 | | | 30.5 | 842 | | 34.0 | 1832 | | 32.8 | 2164 | | 38.1 | | 22384 | | 37.2 | |  |
| Metropolitan | | 46132 | | 48.3 | | 11330 | | | 52.4 | 1011 | | 40.8 | 2773 | | 49.7 | 2538 | | 44.6 | | 28480 | | 47.4 | |  |
| Level of ED | |  | |  | |  | | |  |  | |  |  | |  |  | |  | |  | |  | | <0.001 |
|  | Level 1 | 31601 | | 33.1 | | 6856 | | | 31.7 | 745 | | 30.0 | 2045 | | 36.7 | 1736 | | 30.5 | | 20219 | | 33.6 | |  |
|  | Level 2 | 48547 | | 50.8 | | 11417 | | | 52.8 | 1227 | | 49.5 | 2801 | | 50.2 | 2939 | | 51.7 | | 30163 | | 50.1 | |  |
|  | Level 3 | 15361 | | 16.1 | | 3345 | | | 15.5 | 508 | | 20.5 | 733 | | 13.1 | 1010 | | 17.8 | | 9765 | | 16.2 | |  |
| Mental status at ED entrance | | |  | |  | |  |  | | |  | | |  | | |  | |  |  |  | |  | <0.001 |
|  | Alert | 38467 | | 40.3 | | 11749 | | | 54.3 | 1246 | | 50.2 | 2609 | | 46.8 | 2786 | | 49.0 | | 20077 | | 33.4 | |  |
|  | Verbal response | 15344 | | 16.1 | | 2528 | | | 11.7 | 247 | | 10.0 | 890 | | 16.0 | 717 | | 12.6 | | 10962 | | 18.2 | |  |
|  | Pain response | 15262 | | 16.0 | | 1296 | | | 6.0 | 218 | | 8.8 | 592 | | 10.6 | 496 | | 8.7 | | 12660 | | 21.0 | |  |
|  | Unresponsiveness | 11157 | | 11.7 | | 2667 | | | 12.3 | 257 | | 10.4 | 756 | | 13.6 | 693 | | 12.2 | | 6784 | | 11.3 | |  |
|  | Unknown | 15279 | | 16.0 | | 3378 | | | 15.6 | 512 | | 20.6 | 732 | | 13.1 | 993 | | 17.5 | | 9664 | | 16.1 | |  |
| Length of stay, hour | |  | |  | |  | | |  |  | |  |  | |  |  | |  | |  | |  | | <0.001 |
|  | 0~1 | 8786 | | 9.2 | | 1809 | | | 8.4 | 137 | | 5.5 | 211 | | 3.8 | 354 | | 6.2 | | 6275 | | 10.4 | |  |
|  | 2~4 | 42050 | | 44.0 | | 7760 | | | 35.9 | 1149 | | 46.3 | 2207 | | 39.6 | 2791 | | 49.1 | | 28143 | | 46.8 | |  |
|  | 5~8 | 22765 | | 23.8 | | 5663 | | | 26.2 | 630 | | 25.4 | 1614 | | 28.9 | 1466 | | 25.8 | | 13392 | | 22.3 | |  |
|  | 9~ | 21886 | | 22.9 | | 6382 | | | 29.5 | 564 | | 22.7 | 1543 | | 27.7 | 1074 | | 18.9 | | 12323 | | 20.5 | |  |
|  | Median, IQR | 3.7 (2.0-7.4) | | | | 4.7 (2.4-9.3) | | | | 3.9 (2.3-7.4) | | | 4.7 (2.6-8.8) | | | 3.7 (2.2-6.5) | | | | 3.4 (1.8-6.7) | | | | <0.001 |
| Life-sustaining treatment in the ED | | | | |  | |  |  | | |  | | |  | | |  | |  |  |  | |  |  |
|  | CPR | 20585 | | 21.6 | | 1795 | | | 8.3 | 331 | | 13.3 | 1026 | | 18.4 | 924 | | 16.3 | | 16509 | | 27.4 | | <0.001 |
|  | Intubation | 28780 | | 30.1 | | 2936 | | | 13.6 | 592 | | 23.9 | 1804 | | 32.3 | 1412 | | 24.8 | | 22036 | | 36.6 | | <0.001 |

IQR, interquartile range; ED, emergency department; CPR, cardiopulmonary resuscitation
